# Evaluating Surveillance for Excessive Alcohol Use in New Mexico

**DOI:** 10.5888/pcd15.180358

**Published:** 2018-12-20

**Authors:** Abby Hagemeyer, Alejandro Azofeifa, Donna F. Stroup, Laura E. Tomedi

**Affiliations:** 1 Council of State and Territorial Epidemiologists Applied Epidemiology Fellowship Program, Atlanta, Georgia; 2 Independent researcher, Washington, District of Columbia; 3 Data for Solutions, Inc, Decatur, Georgia; 4 Epidemiology and Response Division, New Mexico Department of Health, Santa Fe, New Mexico

## Abstract

**Purpose and Objectives:**

Prevalence of excessive alcohol use and alcohol-attributable mortality is much higher in New Mexico than in other US states. In 2010, excessive alcohol use cost the state roughly $2.2 billion. Moreover, age-adjusted deaths from alcohol-related chronic liver disease increased 52.5% from 14.1 cases in 2010 to 21.5 cases in 2016. In 2017, the New Mexico Department of Health piloted the Recommended Council of State and Territorial Epidemiologists (CSTE) Surveillance Indicators for Substance Abuse and Mental Health, using 5 indicators to monitor alcohol use and health consequences. The purpose of this study is to evaluate the alcohol surveillance system implemented in New Mexico to ensure that the system yields useful, timely data that can help create effective public health interventions and that resources required for surveillance are adequate.

**Intervention Approach:**

CSTE alcohol surveillance system data come from existing national and state-based surveys and vital statistics.

**Evaluation Methods:**

This evaluation assessed attributes defined in Evaluating Behavioral Health Surveillance Systems and Centers for Disease Control and Prevention guidelines for evaluating public health surveillance systems. Assessment was informed through data collection, systematic literature review searches, and an interview with the alcohol epidemiologist at New Mexico Department of Health.

**Results:**

The CSTE alcohol surveillance system in New Mexico is a useful, stable, and accepted system with good representativeness and population coverage. Data sharing and collaboration between centers within New Mexico Department of Health are well-established, making data access easy and timely. Lastly, the resources required for data collection are accountable and adequate.

**Implications for Public Health:**

The CSTE alcohol surveillance system brings together information (alcohol consumption behaviors and associated morbidity, mortality, and policy-related measures) necessary to show a clear picture of the alcohol effects in New Mexico. This information yields useable, timely data from which the state can monitor trends and develop interventions to reduce the prevalence of alcohol-attributable morbidity and mortality.

## Introduction

In the United States, excessive alcohol use accounts for more than 80,000 deaths annually, making it the third leading cause of preventable death (1) and a significant contributor to alcohol-related injury, disease, and death. Binge drinking, the most frequent and deadly type of excessive alcohol use (1–3), costs the US nearly $200 billion annually, including decreased workplace productivity losses and health care and criminal justice expenses (4). In 2016, the US age-adjusted liver disease and cirrhosis mortality rate per 100,000 people was 10.8, ranging from 6.7 (Maryland) to 24.9 (New Mexico) (5).

New Mexico has disproportionately higher excessive alcohol use and alcohol-attributable deaths compared with other US states. In 2010, excessive alcohol use cost the state roughly $2.2 billion (4). New Mexico has the highest age-adjusted alcohol-attributable death rate in the nation (1). The age-adjusted rate of alcohol-related chronic liver disease deaths in New Mexico increased 52.5% from 14.1 cases in 2010 to 21.5 cases in 2016, making it the leading cause of alcohol-attributable death in the state (6).

In response to substance abuse and mental health problems in the nation, the Council of State and Territorial Epidemiologists (CSTE) established a workgroup to develop behavioral health indicators in the domains of alcohol, other drugs, and mental health using a consensus methodology (7,8). After consensus by stakeholders at the 2016 CSTE Annual Conference, CSTE members recommended regular collection of the 18 indicators to measure and monitor substance abuse and mental health (SA/MH indicators) in state, territorial, local, and tribal surveillance. While many indicators already may be examined in a piecemeal fashion, consensus indicators provide an integrated view of the burden of behavioral health conditions, comparable across time and across jurisdictions (8).

On June 15, 2016, CSTE released a request for proposals for state and local jurisdictions to pilot the SA/MH indicators. The New Mexico Department of Health (NMDOH) was subsequently awarded funding from CSTE to implement the collection and reporting of key surveillance indicators to monitor alcohol use and other related measures: 1) adult binge drinking, 2) youth binge drinking, 3) alcohol-related crash deaths, 4) liver disease and cirrhosis mortality, and 5) state alcohol excise tax, herein collectively referred to as the CSTE alcohol surveillance system. Data collection took place from January to June 2017.

Evaluating public health surveillance systems is critical to ensure that resulting data are timely and useful for actionable public health interventions and that resources required for surveillance are adequate (9). Existing guidelines for evaluating public health surveillance systems are used generally for infectious diseases but present challenges for use with behavioral health surveillance systems (10,11). To address this gap, CSTE formed a workgroup to revise and adapt the existing guidelines to evaluate behavioral health surveillance systems (9). These revisions, along with guidelines from the Centers for Disease Control and Prevention (CDC) (10,11) were used to qualitatively and quantitatively evaluate the CSTE alcohol surveillance system implemented in New Mexico. The evaluation presented here took place from October through December 2017.

## Purpose and Objectives

Factors considered in the development of the SA/MH indicators are published in Recommended CSTE Surveillance Indicators for Substance Abuse and Mental Health (7,8). The purpose of this study was to evaluate the CSTE alcohol surveillance system implemented in New Mexico to ensure that the system yields useful, timely data that can help create effective public health interventions and that resources required for surveillance are adequate.

## Intervention Approach

The recommended CSTE surveillance indicators for SA/MH are designed for state-level data collection and draw from 7 existing data sources. Ultimately, all states will collect and report uniform data based on the specified indicator definitions and methods (7,8). The goal of this national surveillance system is to facilitate sharing data between public health authorities at the state and federal level, with stakeholders, and with the public. These data can be used to inform prevention and control and to evaluate public health programs. This article reports on an evaluation of the CSTE alcohol surveillance system implemented in New Mexico, based on data from the Behavioral Risk Factor Surveillance System (BRFSS) (2014), the Youth Risk Behavior Surveillance System (YRBS) (2015), the Fatality Analysis Reporting System (FARS) (2014), New Mexico vital records (2014), and the Alcohol Policy Information System (APIS) (2016).

### Surveillance system description

CDC’s BRFSS is an annual cross-sectional telephone survey that collects health risk behavior information from US resident adults aged 18 years or older (12,13). Several alcohol measures are captured in the survey, including CSTE’s alcohol surveillance indicator *adult binge drinking,* defined as men having 5 or more drinks on 1 occasion and women having 4 or more drinks on 1 occasion.

The YRBS is national, state, territorial, tribal, and local school-based cross-sectional surveys that collect information on 6 areas of priority health-risk behaviors among high school youths (14). Several other alcohol measures are captured in the survey, including CSTE’s alcohol surveillance indicator *youth binge drinking,* defined (before 2017) as 5 or more drinks of alcohol in a row, within a couple of hours (15).

The National Highway Traffic Safety Administration (NHTSA) maintains FARS, a standardized web database containing data from motor vehicle crashes that occur on public traffic ways resulting in at least 1 death within 30 days from the crash (16,17). FARS provides information on several alcohol-related measures, including CSTE’s alcohol surveillance indicator *alcohol-related crash deaths* by highest blood alcohol concentration (BAC) in the crash and highest driver BAC in the crash. Highest BAC in the crash is defined as the highest BAC recorded among tested individuals involved in the crash, including drivers and nonmotorists (eg, pedestrians, bicyclists); highest driver BAC in the crash is defined as the highest BAC recoded among tested drivers (16).

According to New Mexico State Statute, a funeral service practitioner has the responsibility to obtain demographic data from the next of kin, obtain the medical certificate of cause of death, and file the death certificate for deaths occurring in the state (18). A death certificate for each death in the state is stored with the NMDOH, Epidemiology and Response Division, Bureau of Vital Records and Health Statistics. For New Mexico residents who die outside of the state, death certificate data can be obtained through the State and Territorial Exchange of Vital Events (STEVE). These data include information on several alcohol-related death measures, including CSTE’s alcohol surveillance indicator *liver disease and cirrhosis mortality,* defined as an underlying cause of death with an *International Statistical Classification of Diseases and Related Health Problems* (ICD-10) code of K70.x, K73.x, or K74.x.

APIS, sponsored by the National Institute of Alcohol Abuse and Alcoholism (NIAAA), is updated annually to provide information on 33 alcohol-related policies in the United States (19), including CSTE’s alcohol surveillance indicator *state alcohol excise tax*, reported for beer, wine, and spirits. Specific excise taxes are taxes charged per gallon either at the wholesale or retail level. An exception to reporting is made when a state acts as a control state, meaning that the “state sets the prices of and gains direct profit from wholesale and/or retail off-premises sales” (20).

## Evaluation Methods

For pilot collection and reporting of the SA/MH indicators, the CSTE alcohol surveillance system was implemented in New Mexico and evaluated by using attributes defined in CSTE and CDC guidelines (9,10), using both qualitative and quantitative evaluation methods. Because the data for the CSTE alcohol surveillance system come from existing data sources, attributes are a function of those data sources. In these instances, each data source will be discussed alongside the CSTE alcohol surveillance system. Using both evaluation frameworks (9,10), system attributes were assessed as follows:

1. Usefulness was assessed by determining whether the system describes the public health impact of alcohol consumption at the state level and how that information assists the NMDOH in prevention and intervention efforts. Specifically, we calculated the average scores of perceived usefulness of each alcohol indicator, based on a scale of 1 to 5, from a structured interview with the alcohol epidemiologist in New Mexico.
2. Simplicity was assessed by investigating how the NMDOH collects and accesses the data for each alcohol indicator.
3. Stability was assessed for each alcohol indicator based on the respective system’s operations availability and reliability in New Mexico, including the ability to collect, manage, and produce data useful to inform interventions.
4. Flexibility was assessed by determining how easily the CSTE alcohol surveillance system adjusts to a new demand (ie, adding or modifying a question).
5. Data quality was assessed in terms of the validity and completeness of data, reported in the existing literature, for each alcohol indicator in New Mexico.
6. Acceptability was assessed in terms of persons’ and groups’ willingness to participate in data collection and reporting for the CSTE alcohol surveillance system.
7. Representativeness was assessed by determining if the population under surveillance is representative of the overall population in New Mexico at risk for the respective behavior, risk factor, or health event.
8. Population coverage was assessed by determining if the population under surveillance accurately describes the base population the system was designed to survey.
9. Timeliness was assessed by the amount of time it took the NMDOH to access the appropriate database, abstract and process the data, and produce interpretation and report.

Data from 4 of the 5 alcohol indicators — adult binge drinking, youth binge drinking, alcohol-related crash deaths, and state excise tax — were accessed and collected according to specifications in Recommended CSTE Surveillance Indicators for Substance Abuse and Mental Health (7,8). Information from published literature was used to analyze the flexibility, data quality, acceptability, representativeness, stability, and population coverage of each alcohol indicator. Additionally, a structured interview with the alcohol epidemiologist at the NMDOH was conducted to inform much of the previously described attribute assessments for the liver disease and cirrhosis mortality indicator and the overall CSTE alcohol surveillance system.

## Results

### Usefulness

The CSTE alcohol surveillance system implemented in New Mexico combines key indicators collected from various data sources, described previously, to assess alcohol measures at the state level. The system incorporates morbidity, mortality, and policy-related indicators to help paint a more complete picture of the alcohol-attributable burden in New Mexico, of which the piecewise indicators are incapable. The alcohol indicator system helps estimate the magnitude of behavioral health measures such as binge drinking (adults and youths) as well as the morbidity and mortality of excessive alcohol consumption in the population. Additionally, this surveillance system may enable detection of trends in excessive alcohol use and the morbidity and mortality associated with excessive alcohol use. Moreover, it will be possible to assess how alcohol-related policy, specifically state excise tax, may affect excessive alcohol consumption.

Results from the structured interview in New Mexico determined that the usefulness of the CSTE alcohol surveillance system rated 4.4, where 1 is not useful and 5 is very useful. The individual indicators were rated as follows: adult binge drinking, youth binge drinking, and liver disease and cirrhosis mortality rate were rated 5, while state excise tax was rated 4 and alcohol-related crash deaths was rated 3. Unfortunately, APIS does not report excise tax for microbreweries in New Mexico, which is an important aspect to consider. In New Mexico, the excise tax on beer is $0.41 per gallon, with the exception of beer produced in microbreweries that are taxed at $0.08 per gallon for the first 310,000 gallons and $0.28 per gallon thereafter (21). The indicator for alcohol-related crash deaths, as written in 2016 (original document not available) was not a useful indicator because the definition was confusing and the desired information was difficult to locate in FARS. The NMDOH reports these collected indicators in New Mexico’s Indicator-Based Information System (NM-IBIS), in community presentations, and in publications including the New Mexico Substance Abuse State Epidemiology Profile and the New Mexico Alcohol Fact Sheet (6,22).

### Simplicity

The CSTE alcohol surveillance system implemented in New Mexico is moderately simple, despite the fact that it uses multiple information sources. Data sources used are routinely collected and reported to public health authorities. Moreover, the intradepartmental relationships that NMDOH has cultivated facilitates data accessibility for binge drinking and liver disease and cirrhosis mortality. On the other hand, the original CSTE definition of alcohol-related crash deaths was not easily interpreted nor obtainable. Terms used in the CSTE definition were not consistent with those published in FARS nor was it possible to directly abstract the indicator from crash fatality reports published in FARS. Rather, the indicator measure would require either 1) downloading a SAS (SAS Institute, Inc) data set and analyzing the data, or 2) accessing the FARS query system, a multistep process that may produce data of poor quality.

### Stability

The CSTE alcohol surveillance system implemented in New Mexico is stable; data come from well-established systems, most of which are federally funded. For example, BRFSS has been used in New Mexico since 1986 (23), and is the gold standard of behavioral health surveillance among adults (12). New Mexico has conducted the Youth Risk and Resiliency Survey (YRRS) as a substitute for the YRBS since 1991 (24,25). In addition to behavioral health questions, the YRRS includes questions that assess resiliency factors (25). CDC provides fiscal and technical support for both the BRFSS and the YRBS/YRRS (26,27), while state coordinators have access to the BRFSS and YRRS data for New Mexico.

Another example is FARS, a very stable system in place since 1975 (16). Data, collected daily by FARS analysts, are used at the local, state, and federal levels as well as within public and private organizations to answer a wide range of questions. Data can be accessed through the FARS query system (1994–2016) or downloaded through NHTSA.

New Mexico death certificate data are also stable, as their collection is written into law. These data are housed within the NMDOH, Epidemiology and Response Division, Bureau of Vital Records and Health Statistics.

Finally, APIS is a stable system, funded by NIAAA and updated yearly. Data are available for most alcohol-related policies since 1998, and state excise tax information is available from 2003 through 2016 (19,28).

### Flexibility

The CSTE alcohol surveillance system implemented in New Mexico is a moderately flexible system. Though all states are expected to eventually adopt and report core alcohol indicators, each state has the autonomy to add indicators that may be particularly important to monitor over time in their jurisdiction. Additionally, CSTE subcommittees and workgroups may choose to add, modify, or omit core indicators.

### Data quality

The data quality of the CSTE alcohol surveillance system implemented in New Mexico is determined by the quality of the individual data sources from which the information is acquired. Questions on the BRFSS assessing alcohol and substance abuse have moderate reliability and validity (29). Binge drinking in particular yields lower but comparable estimates to both the National Health Interview Survey and the National Survey on Drug Use and Health (29). Similarly, a study that assessed the 1999 YRBS concluded that it had good test–retest reliability (30).

However, data quality of FARS is low without correction for missing BAC data that vary by state. In 2016 in New Mexico, 45% of drivers involved in a fatal crash had known BAC test results (31). To address this underreporting, the reports published by NHTSA use a validated multiple imputation method (32). Unfortunately, imputed calculations for BAC data are not included in the FARS query system, yielding results that may be biased. The FARS system provides quality control by using range and consistency checks. Other quality control measures for timeliness, accuracy, and completeness are conducted intermittently. The data quality of death certificate data in New Mexico is high. Death certificates filled out in the state undergo extensive edits for completeness and consistency (18). Additionally, staff who file death certificates are offered training annually. NMDOH is also able to capture data for deaths occurring outside of the state through STEVE (33,34). Lastly, the quality of data on state alcohol excise tax in New Mexico reported on APIS is moderate. While APIS collects and reports tax information and alcohol-related policies nationwide (20), it does not report the subtle differences in state policies. As previously mentioned, New Mexico excise tax for beer produced in microbreweries is unavailable.

### Acceptability

The CSTE alcohol surveillance system has been accepted by NMDOH, 1 of the first 4 states to pilot collection and reporting of the SA/MH indicators, as an integral part of their annual substance abuse and mental health surveillance efforts. NMDOH has collected most of these measures for several years to inform the New Mexico Substance Abuse Epidemiology Profile published annually (22). The NMDOH state epidemiologist and alcohol epidemiologist also participated in the CSTE workgroup that identified and edited the recommended indicators (7).

In addition to assessing the acceptability of the CSTE alcohol surveillance system as a whole, we evaluated the acceptability for each data source (BRFSS, YRRS, FARS, and vital records). Each of these data sources are well-established and accepted systems among participants and/or data collectors and users. The New Mexico BRFSS response rate in 2014 was 52.8% and in 2015 was 52.5%, higher than the US average in both years (47.0% in 2014 and 47.2% in 2015). In 2015, the overall response rate for the New Mexico YRRS was 73%, with a school response rate of 94% and the student response rate of 78% (15). For comparison, the overall response rate for the 2015 national YRBS was 60%, while the school response rate was 69% and the student response rate was 86%. Similarly, FARS data collection and reporting is well established and accepted. State employees, in cooperative agreement with NHTSA, are formally trained as FARS analysts to gather data from state sources and report pertinent information into the standardized FARS web database daily (16). Lastly, the acceptability of death certificate data falls under the New Mexico Statute 24–14–20, which mandates the responsible party and timeline for death certificate reporting.

### Representativeness

The CSTE alcohol surveillance system is representative, based on the representativeness of component data systems. While people without a landline or cellular phone are systematically excluded from the BRFSS, advanced weighting procedures make it representative of the state’s general population (12). State-administered YRBS/YRRS are considered state-representative data for school-aged youths attending public schools (15). This survey does not include information on those youths in juvenile detention centers or private schools or those who are home-schooled. According to the American Community Survey, in 2015 in New Mexico, 92.1% of high school youths attended public school while 7.9% attended a private school. Though FARS does not collect information on fatal crashes on private property, it is still considered representative of the overall state population (35). Lastly, New Mexico death certificate data are representative. Not only does a death certificate have to be completed for each death in the state, STEVE allows NMDOH to access death certificate data for residents who died outside of the state.

### Population coverage

The CSTE alcohol surveillance system has good population coverage, based on the population coverage from each data system it involves. The target population of the BRFSS is all resident adults aged 18 years or older with a landline or cellular telephone in the state. While refusal to participate may affect the population coverage, response rates for the survey are respectable. In regard to population coverage, the YRBS/YRRS cannot capture information on students who were absent on the day of the survey or on students who did not receive parental consent to participate. Additionally, youths who dropped out of public school are also missed by this survey; NM-IBIS indicates that the 2015–2016 graduation rate was 71%. High school dropouts and youths who skipped school may have a disproportionately higher affinity to binge drink given their risk behaviors (36). Additionally, population coverage might be affected if schools do not agree to participate in the YRBS/YRRS nonrandomly. The FARS database is a census of fatal traffic crashes occurring on public traffic ways in the United States, collected at the state level daily. Although unlikely, a fatal crash may not be reported. New Mexico death certificate data have good population coverage; a death certificate is completed for each death in the state. New Mexico also has access to death certificates for residents who died elsewhere. The system would miss only deaths that go unreported, which is unlikely.

### Timeliness

The CSTE alcohol surveillance system implemented in New Mexico is moderately timely in the sense that most of the data can be collected quickly once they become available from the respective data sources. Data for binge drinking may be abstracted directly through the corresponding CDC webpage (BRFSS for adults [37], YRBS/YRRS for youths [38]) or accessed by requesting record level access from the New Mexico BRFSS and YRRS coordinators. Collecting information directly from the CDC webpage takes approximately 30 minutes. However, a long lag may occur among data collection, analysis, and final reports; for example, data collected in 2015 are released in the fall of 2016. Alternatively, after data collection and cleaning, record-level access can be requested from the respective coordinator. In New Mexico, data access is usually granted from the corresponding data steward within 1 hour of request. Subsequent analyses and reporting of BRFSS and YRRS would take approximately 1 hour.

Similarly, alcohol-related crash deaths and the liver disease and cirrhosis mortality rate can be reported from either a query system or record-level data. While the FARS query can be completed in a fraction of the time with respect to record-level data, users are advised to interpret results with caution because of missing BAC data. Unfortunately, downloading FARS data, which imputes missing BAC data, to conduct analyses would be time intensive as the user would need to become familiar with the FARS data set, the multiple imputation methods used, and SAS coding. The liver disease and cirrhosis mortality rate can be reported from the NM-IBIS (unfortunately, the latest data available in the query system are from 2013) or through CDC WONDER (CDC Wide-ranging Online Data for Epidemiologic Research), which too has limitations including timeliness and granularity. Alternatively, the measure can be reported directly from death certificate data. The NMDOH, Epidemiology and Response Division, Bureau of Vital Records and Health Statistics, houses New Mexico death certificate data. Once a data request is made, full record-level access is usually granted within 1 day. Subsequent analysis and reporting would take approximately 2 hours.

## Implications for Public Health

While existing data are used for New Mexico’s alcohol surveillance system, the CSTE system concurrently monitors behavioral health measures (binge drinking), policy measures (state alcohol excise tax), and the associated adverse consequences (alcohol-related crash deaths and liver disease and cirrhosis mortality) in a new context. Viewing these indicators together provides a more complete and holistic understanding of the public health impact of excessive alcohol consumption and its health consequences. Relationships among the indicators may become apparent (eg, the association of excise taxes and binge drinking). In addition, the 2 evaluation frameworks used (9,10) aid in the understanding of this complex behavioral context in New Mexico, thus facilitating the development of effective interventions.

Data sharing and collaboration between centers within the NMDOH and among federal partners are well established. These longstanding relationships have made the collection and reporting of the CSTE alcohol surveillance system feasible in New Mexico. Therefore, the amount of time and resources needed to collect this information are minimal and adequate. These indicators will yield useable and timely data from which the state can monitor trends and develop interventions if necessary. Anecdotally, state behavioral health departments and bureaus in the United States often work independently from health departments, making it difficult to understand behavioral health as whole. The use of the SA/MH indicators represents a great example of how to integrate existing behavioral health systems. These integrations aid the understanding of the complex nature of alcohol behaviors in New Mexico, and results could drive interventions to decrease alcohol-attributable deaths in New Mexico.

Through this evaluation, we learned that the CSTE definition for alcohol-related crash deaths needs to be redefined. The first version of the CSTE definition, published in 2016, did not use terms consistent with those from NHTSA nor was the requested information easily reported. As a result of this evaluation, the definition was updated in the second version of the Recommended CSTE Surveillance Indicators for Substance Abuse and Mental Health, published in 2017 (7).

This evaluation is informed through the data collection process, interviews, and published literature and is the first evaluation study to use the newly published Evaluating Behavioral Health Surveillance Systems (9), which reframes existing public health surveillance system evaluation criteria for evaluation of behavioral health surveillance. In behavioral health surveillance, sensitivity is closely related to completeness of data (ie, data quality attribute) (9), therefore sensitivity was not directly assessed in this evaluation. Although the new revised evaluation framework (9) provides alternative ways to evaluate sensitivity, this was not possible because of the lack of a gold standard.

The indicators included ICD-10 codes K70.x, K73.x, and K74.x; however, only K70.x codes are alcohol-related. The K73 and K74 codes are specified as not alcohol-related causes of death, with the exception of K74.6 and K73.9 which are “unspecified causes.” This might result in an over-estimation of liver and cirrhosis mortality related to alcohol use. CSTE will examine this potential bias in any upcoming revision of the indicators.

Of the 9 items in the evaluation framework, only the usefulness indicator was scored because of its nature of being objective and interpretable. In future evaluation activities, scoring will be investigated for other system attributes. The evaluation of timeliness concerned the time needed to retrieve, analyze, and interpret the data and is closely related to feasibility. Because of use of existing data, time lag and year of occurrence may differ among data sources.

Liver disease is the only mortality indicator represented in the alcohol surveillance system evaluated here; one objective of indicator selection was to include a range of data sources and look at various types of impact (morbidity and mortality) for alcohol, drugs, and mental illness or self-harm, while balancing the burden of reporting. Among the 18 indicators (7), few rely on death data. In addition, the system does not include measurement of morbidity indicators to represent the cost of a chronic condition.

New Mexico’s intradepartmental relationships and data sharing practices could serve as a model for other states. This evaluation did not address feasibility and cost of implementation in jurisdictions not using these indicators because this evaluation was conducted by a health department that was 1) involved in developing the indicators and 2) already collecting or using most of these indicators. An ongoing economic evaluation of 15 states piloting the indicators indicates that New Mexico’s cost was high (about $1,110 per year) compared with the overall range of $20.24 to $4,065.78, an average of $697. While it is true that New Mexico was involved in the development of the indicators, staff who participated in the evaluation were not involved in development; furthermore, all states were thoroughly involved with ratification and adoption of the indicators. Thus, it would not have been possible to conduct the evaluation in a state totally blinded to indicator development.

## References

[1] Stahre M , Roeber J , Kanny D , Brewer RD , Zhang X . Contribution of excessive alcohol consumption to deaths and years of potential life lost in the United States. Prev Chronic Dis 2014;11:E109. 10.5888/pcd11.130293 24967831 PMC4075492

[2] Fact sheets — binge drinking. Atlanta (GA): US Department of Health and Human Services, Centers for Disease Control and Prevention, National Center for Chronic Disease Prevention and Health Promotion, Division of Population Health; 2017. https://www.cdc.gov/alcohol/fact-sheets/binge-drinking.htm. Accessed September 20, 2018.

[3] Esser MB , Hedden SL , Kanny D , Brewer RD , Gfroerer JC , Naimi TS . Prevalence of alcohol dependence among US adult drinkers, 2009–2011. Prev Chronic Dis 2014;11:E206. 10.5888/pcd11.140329 25412029 PMC4241371

[4] Sacks JJ , Gonzales KR , Bouchery EE , Tomedi LE , Brewer RD . 2010 National and state costs of excessive alcohol consumption. Am J Prev Med 2015;49(5):e73–9. 10.1016/j.amepre.2015.05.031 26477807

[5] Chronic liver disease/cirrhosis mortality by state. Atlanta (GA): US Department of Health and Human Services, Centers for Disease Control and Prevention, National Center for Health Statistics; 2017. https://www.cdc.gov/nchs/pressroom/sosmap/liver_disease_mortality/liver_disease.htm. Accessed September 20, 2018.

[6] New Mexico Department of Health. Alcohol use in New Mexico. https://ibis.health.state.nm.us/indicator/view/AlcoholRelatedDthLiver.Year.NM_US.html. Accessed September 20, 2018.

[7] Council of State and Territorial Epidemiologists. Recommended CSTE surveillance indicators for substance abuse and mental health. Atlanta (GA): Council of State and Territorial Epidemiologists, Substance Use and Mental Health Subcommittee (2017 revision); 2017. http://c.ymcdn.com/sites/www.cste.org/resource/resmgr/pdfs/pdfs2/2017RecommenedCSTESurvIndica.pdf. Accessed September 20, 2018.

[8] Hopkins RS , Landen M , Toe M . Development of indicators for public health surveillance of substance use and mental health. Public Health Rep 2018;133(5):523–31. 10.1177/0033354918784913 30075094 PMC6134563

[9] Azofeifa A , Stroup DF , Lyerla R , Largo T , Gabella BA , Smith CK , Evaluating behavioral health surveillance systems. Prev Chronic Dis 2018;15:E53. 10.5888/pcd15.170459 29752804 PMC5951155

[10] German RR , Lee LM , Horan JM , Milstein RL , Pertowski CA , Waller MN . Updated guidelines for evaluating public health surveillance systems: recommendations from the Guidelines Working Group. MMWR Recomm Rep 2001;50(RR-13):1–35, quiz CE1–7. https://www.cdc.gov/mmwr/preview/mmwrhtml/rr5013a1.htm 18634202

[11] Groseclose S , German R , Nsbuga P . Evaluating public health surveillance. In: Lee L, Teutsch S, Thacker S, Louis MS, editors. Principles and practice of public health surveillance. Third edition. New York (NY): Oxford University Press; 2010. p.166-197.

[12] Centers for Disease Control and Prevention. Behavioral Risk Factor Surveillance System overview: BRFSS 2016. Altanta (GA): US Department of Health and Human Services; 2017. https://www.cdc.gov/brfss/annual_data/2016/pdf/overview_2016.pdf. Accessed September 20, 2018.

[13] Behavioral Risk Factor Surveillance System: about BRFSS. Atlanta (GA): US Department of Health and Human Services, Centers for Disease Control and Prevention, National Center for Chronic Disease Prevention and Health Promotion, Division of Population Health; 2017. https://www.cdc.gov/brfss/about/index.htm. Accessed September 20, 2018.

[14] Brener ND , Kann L , Shanklin S , Kinchen S , Eaton DK , Hawkins J , Methodology of the Youth Risk Behavior Surveillance System — 2013. MMWR Recomm Rep 2013;62(RR-1):1–20. https://www.cdc.gov/mmwr/pdf/rr/rr6201.pdf. 23446553

[15] Kann L , McManus T , Harris WA , Shanklin SL , Flint KH , Hawkins J , Youth Risk Behavior Surveillance — United States, 2015. MMWR Surveill Summ 2016;65(6):1–174. 10.15585/mmwr.ss6506a1 27280474

[16] National Center for Statistics and Analysis. Fatality Analysis Reporting System: brochure. Washington (DC): National Highway Traffic Safety Administration; 2014. Accessed September 20, 2018.

[17] Fell JC , Tippetts AS , Voas RB . Fatal traffic crashes involving drinking drivers: what have we learned? Ann Adv Automot Med 2009;53:63–76. https://www.ncbi.nlm.nih.gov/pmc/articles/PMC3256806/ 20184833 PMC3256806

[18] Indicator-Based Information System for Public Health. Santa Fe (NM): New Mexico Department of Health; 2017. http://ibis.health.state.nm.us. Accessed September 20, 2018.

[19] Hilton M . APIS: the NIAAA Alcohol Policy Information System. Alcohol Res 2013;35(2):184–5. https://www.ncbi.nlm.nih.gov/pmc/articles/PMC3908709/ 24881326 10.35946/arcr.v35.2.08 PMC3908709

[20] Alcohol Policy Information System. Bethesda (MD): US Department of Health and Human Services, National Institute on Alcohol Abuse and Alcoholism; 2017. https://www.alcoholpolicy.niaaa.nih.gov. Accessed September 20, 2018.

[21] New Mexico Taxation and Revenue Department. Notice: 2014 liquor excise tax return rate changes. Santa Fe (NM): 2014. RPD-NTC 651.13.

[22] New Mexico Department of Health. New Mexico substance abuse epidemiology profile. Santa Fe (NM): 2017. https://nmhealth.org/data/view/substance/2067/. Accessed September 20, 2018.

[23] Landen MG , Ringwalt CL , Rosenblatt T , Zigich L , Honey WA , Anderson L . Health behaviors and conditions of adult New Mexicans 2013: results from the New Mexico Behavioral Risk Factor Surveillance System (BRFSS). Santa Fe (NM): New Mexico Department of Health; 2014. https://nmhealth.org/data/view/behavior/1468/. Accessed September 20, 2018.

[24] Centers for Disease Control and Prevention. YRBS participation history, data quality, and data availability. Atlanta (GA): US Department of Health and Human Services; 2016. https://www.cdc.gov/healthyyouth/data/yrbs/pdf/2015/2015_hs_participation_history.pdf. Accessed September 20, 2018.

[25] Green D , Penaloza LJ , Chrisp E , Dillon M , Cassell CM , Tsinajinnie E , New Mexico Youth Risk and Resiliency Survey (YRRS): 2005 report of state results. Santa Fe (NM): New Mexico Departments of Health and New Mexico Public Education Department; 2006. https://files.eric.ed.gov/fulltext/ED500394.pdf. Accessed September 20, 2018.

[26] Centers for Disease Control and Prevention. Behavioral Risk Factor Surveillance System: history. Atlanta (GA): US Department of Health and Human Services; 2012. https://www.cdc.gov/brfss/factsheets/pdf/brfss-history.pdf. Accessed September 20, 2018.

[27] YRBSS frequently asked questions. Atlanta (GA): US Department of Health and Human Services, Centers for Disease Control and Prevention, National Center for HIV/AIDS, Viral Hepatitis, STD, and TB Prevention, Division of Adolescent and School Health; 2017. https://www.cdc.gov/healthyyouth/data/yrbs/faq.htm. Accessed September 20, 2018.

[28] Bloss G . The Alcohol Policy Information System (APIS) and policy research at NIAAA. Alcohol Res Health 2011;34(2):246–7. https://www.ncbi.nlm.nih.gov/pmc/articles/PMC3860562/ 22330224 PMC3860562

[29] Pierannunzi C , Hu SS , Balluz L . A systematic review of publications assessing reliability and validity of the Behavioral Risk Factor Surveillance System (BRFSS), 2004–2011. BMC Med Res Methodol 2013;13(1):49. 10.1186/1471-2288-13-49 23522349 PMC3622569

[30] Brener ND , Kann L , McManus T , Kinchen SA , Sundberg EC , Ross JG . Reliability of the 1999 Youth Risk Behavior Survey questionnaire. J Adolesc Health 2002;31(4):336–42. 10.1016/S1054-139X(02)00339-7 12359379

[31] National Center for Statistics and Analysis. Traffic safety facts. 2016 Data. State alcohol-impaired-driving estimates. Washington (DC): National Highway Traffic Safety Administration; 2018. (DOT HS 812 483). https://crashstats.nhtsa.dot.gov/Api/Public/ViewPublication/812483. Accessed September 20, 2018.

[32] National Center for Statistics and Analysis. Traffic safety facts. 2016 Data. Alcohol-impaired driving. Washington (DC): National Highway Traffic Safety Administration; 2017. (DOT HS 812 450). https://crashstats.nhtsa.dot.gov/Api/Public/ViewPublication/812450. Accessed September 20, 2018.

[33] Schwartz S . The U.S. Vital Statistics System: the role of state and local health departments. 2009. https://www.ncbi.nlm.nih.gov/books/NBK219870/. Accessed November 25, 2018.

[34] National Association for Public Health Statistics and Information Systems. Information systems for vital records stewardship. https://www.naphsis.org/systems. Accessed November 25, 2018.

[35] Fatality Analysis Reporting System (FARS) encyclopedia. Washington (DC): US Department of Transportation, National Highway Traffic Safety Administration, National Center for Statistics and Analysis; 2017. https://www-fars.nhtsa.dot.gov/Help/Terms.aspx. Accessed September 20, 2018.

[36] Townsend L , Flisher AJ , King G . A systematic review of the relationship between high school dropout and substance use. Clin Child Fam Psychol Rev 2007;10(4):295–317. 10.1007/s10567-007-0023-7 17636403

[37] BRFSS prevalence and trends data. Atlanta (GA): US Department of Health and Human Services, Centers for Disease Control and Prevention, National Center for Chronic Disease Prevention and Health Promotion, Division of Population Health; 2017. https://www.cdc.gov/brfss/brfssprevalence/index.html. Accessed September 20, 2018.

[38] High school YRBS. Atlanta (GA): US Department of Health and Human Services, Centers for Disease Control and Prevention; 2017. https://nccd.cdc.gov/youthonline/App/Default.aspx. Accessed September 20, 2018.

